# Associations between grip strength, cardiorespiratory fitness, cardiovascular risk and mental health in forcibly displaced people from a Greek refugee camp

**DOI:** 10.1038/s41598-023-48032-5

**Published:** 2023-11-28

**Authors:** Markus Gerber, Konstantinia Filippou, Florian Knappe, Ioannis D. Morres, Emmanouil Tzormpatzakis, Elsa Havas, Harald Seelig, Flora Colledge, Sebastian Ludyga, Marianne Meier, Yannis Theodorakis, Roland von Känel, Uwe Pühse, Antonis Hatzigeorgiadis

**Affiliations:** 1 Department of Sport, Exercise and Health, University of Basel, Grosse Allee 6, 4052 Basel, Switzerland; 2 Department of Physical Education and Sport Sciences, University of Thessaly, Trikala, Greece; 3 Department of Nutrition and Dietetics, University of Thessaly, Trikala, Greece; 4 Department of Health Sciences and Medicine, University of Lucerne, Lucerne, Switzerland; 5 Interdisciplinary Center for Gender Studies, University of Bern, Bern, Switzerland; 6 Department of Consultation-Liaison Psychiatry and Psychosomatic Medicine, University Hospital Zurich, University of Zurich, Zurich, Switzerland

**Keywords:** Risk factors, Psychology, Human behaviour

## Abstract

Muscular strength represents a specific component of health-related fitness. Hand grip strength is used as a simple and dynamic marker of maximum voluntary force of the hand and to estimate overall strength. Today, little is known about the relationship between grip strength and health in forcibly displaced populations. In the present study, we examined whether grip strength is associated with various health outcomes in a sample of forcibly displaced people living in a Greek refugee camp. The present analyses are part of a larger pragmatic randomized controlled trial. In this paper, cross-sectional baseline data of 143 participants (71 men, 72 women) will be presented. In addition to grip strength, the following physical and mental health outcomes were assessed: body weight and body composition, blood pressure, total cholesterol, low- and high-density lipoprotein cholesterol, triglycerides, blood glucose levels (HbA1c), post-traumatic stress disorder (PTSD) symptoms, depressive and anxiety symptoms, pain, and quality of life. Linear regression analyses were carried out to examine how grip strength is associated with the health outcomes, separately for absolute and normalized grip strength scores. Grip strength was positively and strongly associated with percentage muscle mass (normalized grip strength: Stand. *B* = 0.58, *p* < .001), whereas a negative association existed for percentage body fat (normalized grip strength: Stand. *B* = − 0.58, *p* < .001). No statistically significant associations occurred between grip strength and the other cardiovascular risk markers. In contrast, we found that participants with higher normalized grip strength reported higher levels of PTSD (normalized grip strength: Stand. *B* = 0.36, *p* < .05) and depressive symptoms (normalized grip strength: Stand. *B* = 0.29, *p* < .05). No significant association occurred between grip strength, anxiety, pain and quality of life. Measuring grip strength in forcibly displaced people can be a useful way to assess their overall muscle strength. Grip strength tests are easy to implement, and results can be used to assess the effects of specific intervention measures. Nevertheless, our results question the usefulness of grip strength as a marker of cardiovascular health and mental wellbeing in a refugee camp setting.

## Introduction

Muscular strength represents a specific component of health-related fitness and is defined as the ability to generate maximal muscle force^1^. Hand grip strength is used as a simple and dynamic marker of maximum voluntary force of the hand^2^, and to estimate overall strength^3^. In line with this, grip strength was found to be associated with arm, back and leg strength in adult populations^4^. More generally, poor grip strength may be understood as an indicator of weakness, fatigue, and/or malnutrition, all of which can have negative impacts on a person’s overall health and ability to perform daily tasks. Accordingly, prior research has shown that poor grip strength correlates with a variety of health-related outcomes including nutritional status^5^, activities of daily living^6^, depressive symptoms^7^, impaired health-related quality of life^8^, chronic physical conditions and physical multimorbidity^9^, cancer^10^, cardiovascular diseases^11^, as well as cardiovascular and all-cause mortality^11,12^. Interestingly, muscle strength turned out to be a better predictor of mortality than overall muscle mass^13^. Accordingly, grip strength has the potential to be applied as an inexpensive and easy-to-use risk-stratification method to estimate an individuals’ risk of cardiovascular disease^14,15^.

In the present study, we examined whether grip strength is associated with various health outcomes in a sample of forcibly displaced people living in a Greek refugee camp. As shown in previous research, refugees have a higher risk for mental health issues such as post-traumatic stress disorder (PTSD), depression, and non-communicable diseases^16,17^. Many of them were forcibly displaced due to war, persecution or other forms of violence, and are often left with few resources, little support, and limited access to basic necessities such as food, water and shelter^18^. Refugees often have limited legal rights and protections, and face an increased risk of exploitation and abuse^19^. Today, however, still little is known about the relationship between grip strength and health in forcibly displaced populations. For instance, one study was carried out with older Rwandan refugees living in a refugee camp in Tanzania and showed that poor grip strength is associated with poor nutritional status^20^. In another study, refugees from North Korea were compared with an age-matched sample of controls from South Korea showing that refugees had significantly lower grip strength scores^21^. However, no relationships with other health markers were reported. Thus, while the former study provides some evidence for the association between grip strength and health outcomes among refugees, more research on the relationship between grip strength and health outcomes is needed. Given that access to healthcare is often limited among forcibly displaced people living in refugee camps^18^, easy-to-use screening tools such as the grip strength test could be valuable in identifying people who are at particularly high risk to face physical and mental health challenges.

Given this background, the overall purpose of the present study was to examine whether in a diverse sample of forcibly displaced people living in a refugee camp in Greece, grip strength would be associated with various health outcomes, including cardiorespiratory fitness (VO_2_max), body composition (percentage muscle mass and body fat), blood pressure, blood lipids (cholesterol, triglycerides), blood glucose, symptoms of PTSD, depression and generalized anxiety disorder, pain, and quality of life. To this end, different analyses were performed:

First, we examined associations between absolute grip strength scores and the assessed health measures, after controlling for age, sex, and body mass index (BMI). With regard to age, research has shown that grip strength increases through childhood and adolescence^22,23^ and peaks around the age of 40 years. After that, grip strength declines in a relatively linear way^2^ due to various ageing-related factors such as a decline in muscle mass and strength, changes in joint mobility and flexibility, and changes in the nervous system. With regard to sex, grip strength proved to be higher in men than in women^24^. However, grip strength can vary greatly between individuals of the same sex, and there is considerable overlap between grip strength of men and women^25^. After puberty, there is an exponential progression in muscle strength among boys, which has been ascribed to the increase of testosterone among boys^26^, and the increase of body fat among girls^22,27^.

Second, we examined associations between grip strength and health outcomes after normalization of grip strength scores. Normalization of grip strength is a process that involves adjusting the grip strength measurement to account for individual differences in body weight and height^28,29^. Normalization for participants’ BMI could be relevant because normalized grip strength turned out to be more closely associated with health outcomes than absolute grip strength^30^.

Third, we examined whether participants who fall below or exceed specific grip strength cut-points differ in the assessed health outcomes. While cut-points can vary depending on the population being studied and the purpose of the measurement^2,31^, we used cut-points that have previously been applied in participants in low-to-middle-income countries (LMICs)^9^.

Based on previous investigations, our hypotheses were that men would report higher grip strength than women (Hypothesis 1)^24,25^. We further assumed that the relationship between grip strength and age would be curvilinear with a peak around the age of 40 years (Hypothesis 2)^2^. As strength and cardiovascular fitness reflect different fitness components^32^, we only expected weak positive associations between grip strength and VO_2_max (Hypothesis 3)^33,34^. Regarding the association between grip strength and the various health outcomes, we expected that higher grip strength would be associated with higher muscle mass^35^, lower fat mass^36^, lower cardiovascular risk^37,38^, and better mental health (Hypothesis 4)^7,39^. While we expected similar associations for absolute and normalized grip strength scores^40^, we expected slightly stronger associations for normalized compared to absolute grip strength (Hypothesis 5)^30^.

## Methods

### Participants and procedures

The present analyses are part of a larger pragmatic randomized controlled trial (RCT)^41^. In this paper, cross-sectional baseline data will be presented. Ethical approval was obtained from the local ethical review boards, and all procedures were performed in accordance with the relevant guidelines and regulations, including the ethical principles of the Declaration of Helsinki and the guidelines of Good Clinical Practice (GCP). The study took place in a remote area in Central Greece in a refugee camp, which is under the governance of the Ministry of Migration and Asylum. In the camp, residents live in containers (equipped with a bathroom and kitchen), either together with family members or with max. four people of the same sex and origin. The residents spend most of their time in the camp. Due to legal barriers, they are not allowed to work. Leisure activities are scarce, and the remote location makes it difficult to escape the camp's daily routine.

Participants were eligible if they met the following inclusion criteria: (a) living in the selected refugee camp, (b) 16–59 years old, (c) able to read in English, Arabic, Farsi, or French, and (d) provided written informed consent. The focus of the present study was on older adolescents and adults. Minors (children and younger adolescents < 16 years) were excluded to ensure cognitive understanding of the items included in the psychological questionnaires. Older adults (> 60 years of age) were excluded to facilitate the implementation of the intervention due to decreasing fitness among elderly people^32^. A screening was performed with potentially eligible households to draw a random sample stratified by sex. For the RCT, the minimal estimated sample size was 136 participants to demonstrate an intervention effect on PTSD symptoms^41^.

The screening, recruitment, and data assessment took place in May 2021 by the research team together with 10 trained research assistants who were familiar with the residents' cultural background and the camp's contextual setting. Both written and verbal information were given to the participants in their native languages, and participants provided written informed consent before the first data assessment. All participants were assured that participation is voluntary, and that withdrawal is possible at any time without any disadvantages, particularly with regard to the asylum process. The 10 research assistants were recruited from the camp population and had to be diverse in sociodemographic background (sex and origin) and fluent in English and one of the other languages used in the study. Research assistants received training on the study's purpose and the practical and theoretical elements of data collection and translation (e.g., verbatim translation and confidentiality). Given their familiarity with camp residents’ cultural backgrounds and the camp's contextual environment, the research assistants played an important role in interacting with residents, explaining study procedures, obtaining informed consent, performing translation tasks, and aiding in data collection. Data assessment was carried out at the nearby Department of Physical Education and Sport Science. Participants received information about their results after completion of the assessment and were referred to a specialist in case of a potential health risk. As further incentives, participants received a meal and some sport equipment.

### Measures

For reasons of space, the instruments are only briefly described here. More detailed information can be found in the published study protocol^41^. Table 1 provides information about the internal consistency of the measurements in the present sample.

**Table 1:** Descriptive statistics.

	*N*^a^	*M*	*SD*	*Min*	*Max*	*α*^*b*^	*Skew*	*Kurt*
Sociodemographic background								
Age (in years)	143	29.00	9.32	16	58	–	0.98	0.67
Height (in m)	143	1.64	0.10	1.41	1.86	–	0.15	-0.45
Weight (in kg)	143	69.12	14.97	39.30	117.80	–	0.54	0.34
Body mass index (in kg/m^2^)	143	25.83	5.27	16.79	42.44	–	0.87	0.62
Months away from home country^a^	128	34.68	38.05	2	300	–	4.36	23.40
Months in Koutsochero refugee camp^b^	137	14.66	10.14	0	48	–	0.68	-0.13
Grip strength								
Absolute grip strength (in kg)	143	27.44	9.99	6.50	54.00	0.98	0.28	-0.85
Normalized grip strength	143	1.10	0.46	0.26	2.12	–	0.21	-1.07
Fitness								
Estimated VO_2_max (in mL/kg/min)	121	32.94	12.47	9.05	91.93	–	1.12	3.69
Body composition								
Muscle mass (in %)	142	67.16	10.84	41.23	86.12	–	− 0.27	− 0.94
Fat mass (in %)	142	28.72	11.34	9.30	56.60	–	0.35	− 0.88
Cardiovascular risk markers								
Systolic blood pressure (in mm HG)	142	119.77	13.23	89	156	0.94	0.38	0.02
Diastolic blood pressure (in mm HG)	142	81.28	8.43	59.67	107.67	0.93	0.47	0.37
Total cholesterol (in mmol/l)	126	4.07	0.90	1.92	6.61	–	0.36	-0.14
LDL cholesterol (in mmol/l)	125	2.19	0.70	0.53	3.84	–	0.24	-0.43
HDL cholesterol (in mmol/l)	126	1.19	0.31	0.51	1.95	–	0.38	-0.27
Triglycerides (in mmol/l)	126	1.61	0.80	0.56	5.18	–	1.31	2.57
HbA1c (in %)	132	5.36	0.30	4.70	6.80	–	1.26	3.94
Mental health outcomes								
PTSD symptoms (IES-R)	141	35.21	22.27	0	79	0.94	0.09	− 1.04
Depressive symptoms (PHQ-9)	142	10.92	7.39	0	27	0.87	0.10	− 1.02
Anxiety symptoms (GAD-7)	133	9.50	6.58	0	21	0.90	0.15	− 1.19
Pain (VAS)	131	26.92	21.68	0	95	0.77	0.80	0.18
Quality of life (WHO-5)	141	13.45	7.17	0	25	0.88	− 0.09	1.02

*Notes.* VO_2_max: Maximal oxygen uptake. MVPA: Moderate-to-vigorous physical activity. LDL: Low density lipoprotein. HDL: High density lipoprotein. HbA1c: Glycated hemoglobin. IES-R: 22-item Index of Event Scale—revised. PHQ-9: 9-item depression scale of the Patient Health Questionnaire. GAD-7: 7-item General Anxiety Disorder Index. 7-item ISI: Insomnia Severity Index. VAS: 5-item Visual Analogue Scale. WHO-5: 5-item Quality of Life Index of the World Health Organization. ^a^Variations in *N* due to different number of missings for varying outcomes. ^b^Cronbach’s alpha.

To assess upper-body muscle strength, the Grip Strength Test was applied^42^, using a hydraulic hand dynamometer (Saaehan, Tisselt, Belgium). Participants held their arm in a position at a 90° angle, while being seated and an upright and relaxed position. Participants performed six trials, with 30-s resting periods between trials and alternating between the left and right hand. Data was recorded in kg (to the nearest of 1 kg). The Grip Strength Test has been previously applied in refugee studies^20^. Weak grip strength was defined as < 30 kg for men and < 20 kg for women^9^, using the mean score of all six measurements.

Body weight and body composition were assessed with a digital weighing scale (BC-545, Tanita, USA) that also allowed bioelectrical impedance analysis to measure the percentage of body fat and muscle mass. Body height was assessed with a stadiometer. Blood pressure was assessed with an Omron® digital blood pressure monitor, after participants had rested for 5 min in a seated position^43^. Finger prick methodology was used to obtain capillary blood samples. Total cholesterol, low- (LDL) and high-density (HDL) lipoprotein cholesterol, triglycerides and blood glucose levels (HbA1c) were analysed via Afinion 2 analysers (Abbott, Wädenswil, Switzerland). Afinion 2 point-of-care (PAC) analyser results correspond well with laboratory tests for both lipid levels and HbA1c^44,45^.

All psychological measures were assessed with instruments that have been previously used with forcibly displaced adults^46–49^. Given that many participants had limited English skills, the questionnaires were available in English, Arabic, Farsi, and French language, and translators supported the data assessment process. All instruments have been validated previously in English, Arabic, Farsi, and French^41^, and the scales had acceptable or good internal consistency (Cronbach’s alpha > 0.7) in our pilot study^50^. PTSD symptoms were measured with the 22-item Impact of Event Scale-Revised (IES-R)^51^, which refers to DSM-5^52^ and ICD-10^53^ criteria of PTSD. Answers were given on a five-point Likert scale from 0 (not at all) to 4 (extremely), resulting in an overall index between 0 and 88 points. Depressive symptoms were measured with the 9-item Patient Health Questionnaire (PHQ-9)^54^, which refer to DSM-5 criteria for major depression^52^. Answers were given on a four-point Likert scale from 0 (not at all) to 3 (nearly every day), with overall scores varying between 0 and 27. Anxiety symptoms were measured with the 7-item General Anxiety Disorder scale (GAD-7)^55^, which refers to DSM-5 criteria for generalized anxiety disorder^52^. Answers were given on a four-point Likert scale from 0 (not at all) to 3 (nearly every day), with overall scores ranging between 0 to 21. Pain was measured with the 5-item Visual Analogue Scale for Pain (VAS)^56^, which asked for pain over the last week in the head, back, chest, stomach, arms and legs. Item scores ranged from 0 (no pain) to 100 (pain as bad as it could be) and the average was calculated, to build a total index between 0 and 100. Quality of life was measured with the 5-item World Health Organization (WHO) Index^57^. Answers were given on a Likert scale from 0 (at no time) to 5 (all the time). Items are summed up and multiplied by 4, resulting in an overall index between 0 and 100^58^.

### Statistical analyses

Descriptive statistics (*n*, %, *M*, *SD*, *Min*, *Max*, *Skew*, *Kurt*) are presented to describe the characteristics of the sample and distribution of the study variables. Associations with sex and age were tested via analyses of covariance (ANCOVAs), *chi*^2^-tests, and (linear/quadratic) regression analyses. Linear regression analyses were carried out to examine how grip strength is associated with the health outcomes, separately for absolute and normalized grip strength scores. Age and sex were used as covariates if normalized grip strength was used as independent variable. Beyond age and sex, BMI was considered as additional covariate in the analyses with absolute grip strength as independent variable. Multicollinearity was examined via variance inflation factor (VIF) scores. Two-way ANCOVAs were used to test differences between participants with weak grip strength and their stronger counterparts. In these analyses, grip strength was used as independent variable, and sex was conceptualized as moderating variable. To examine whether sex moderates the relationship between grip strength and the dependent variables, the interaction term was included. Age and BMI were considered as covariates. All analyses were carried out with SPSS version 28 for Mac (IBM Corporation, Armonk, USA), and the level of significance was set at *p* < 0.05 across all analyses.

### Ethics approval and consent to participate

All procedures were approved by the Research Ethics Committee of the University of Thessaly, ref approval no. 39 (date of approval: 19/11/2020), the Ethics Com-mittee of the Department of Physical Education & Sport Science, ref approval no. 1701 (date of approval: 09/12/ 2020) and the ethical review board of Northwest and Central Switzerland, ref approval no. AO_2020-00036 (date of approval: 26/11/2020). Prior to data assessment, all participants signed written informed consent.

## Results

### Sample characteristics

In total, 150 participants (76 men, 74 women) had valid grip strength measurements. However, 7 participants did not have valid data for age, and were therefore excluded from the further data analyses. The remaining sample of 143 participants was composed of 71 men and 72 women, with a mean age of *M* = 29.00 years (*SD* = 9.32) and a mean BMI of *M* = 25.83 kg/m^2^ (*SD* = 5.27) (see Table 1). On average, participants were away from their home country for *M* = 34.68 months (*SD* = 38.05), and lived for *M* = 14.67 months (*SD* = 10.14) in the camp where the current study took place. The following countries of origin were reported *n*: Afghanistan (*n* = 71), Cameroon (*n* = 1), Congo (*n* = 18), Guinea (*n* = 1), Iraq (*n* = 3), Iran (*n* = 8), Pakistan (*n* = 1), Sierra Leone (*n* = 2), Somalia (*n* = 21), Syria (*n* = 12) and Turkey (*n* = 2) (did not answer: *n* = 3). Most of the participants completed the survey in Farsi (*n* = 83), followed by Arabic (*n* = 31), French (*n* = 21) and English (*n* = 8). With regard to educational background, 35 participants reported not to have formal education, 51 completed primary school, 33 high school and 20 completed higher education (university) (did not answer: *n* = 4).

### Descriptive statistics

Table 1 shows the descriptive statistics for all metric study variables. With the exception of one variable (months away from home), none of the variables showed major deviations from normality (defined as Skew > 2 and/or Kurt > 7)^59^. The internal consistency was good for grip strength, blood pressure and across all mental health outcomes.

### Association of grip strength with sex and age

With regard to sex, after controlling for age and BMI, ANCOVAS showed that men (*M* = 35.42 kg, *SD* = 6.95) achieved higher absolute grip strength scores than women (*M* = 19.58 kg, *SD* = 5.04), *F*(1,142) = 245.43, *p* < 0.001, η^2^ = 0.638. A similar sex effect was found for normalized grip strength, after controlling for age, *F*(1,142) = 249.91, *p* < 0.001, η^2^ = 0.641. In line with this, *chi*^2^-analyses revealed that women (43.1%) were more likely to be classified into the group with weak grip strength than men (25.4%), χ^2^(1,143) = 4.97, *p* < 0.05, ϕ = 0.183.

**Figure 1:**
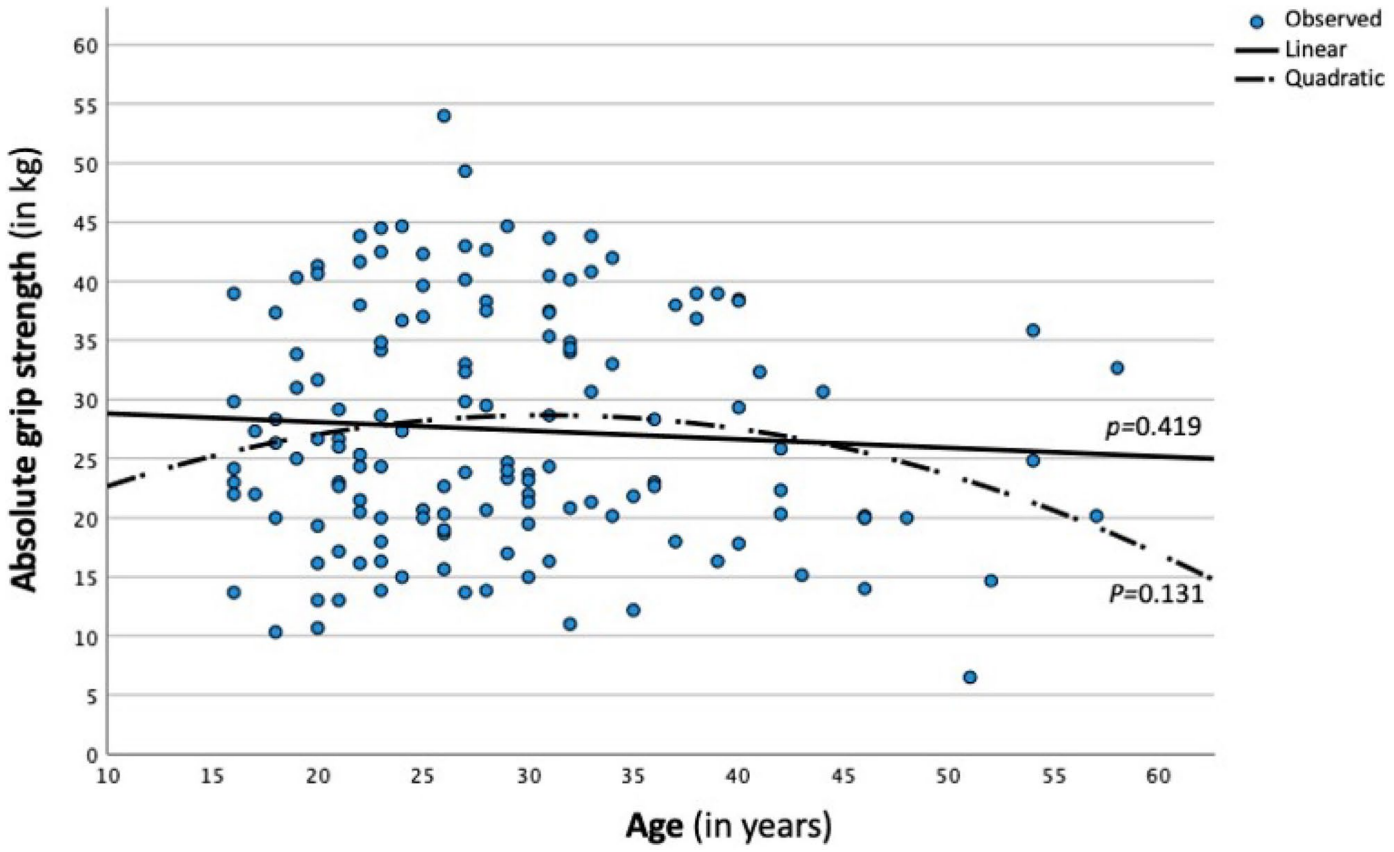
Association between grip strength and age.

With regard to age, after controlling for sex and BMI, linear regression analyses showed that age was neither associated with absolute (β = − 0.068, *p* = 0.231) nor normalized grip strength (β = − 0.076, *p* = 0.131). As shown in Fig. 1, the use of quadratic regression did not result in statistically significant relationships. Finally, after controlling for sex and BMI, participants with weak grip strength (*M* = 27.88 years, *SD* = 9.00) did not differ in age from their stronger counterparts (*M* = 29.59, *SD* = 9.47), *F*(1,142) = 0.17, *p* = 0.685, η^2^ = 0.001.

### Associations between absolute grip strength and health outcomes

Table 2 shows that there was no statistically significant association between absolute grip strength and estimated VO_2_max (β = 0.100, *p* = 0.402). In turn, a positive association was found for percentage muscle mass (β = 0.204, *p* < 0.001), whereas a negative relation existed for percentage body fat (β = -0.208, *p* < 0.001). Absolute grip strength was not statistically significantly associated with any of the cardiovascular risk markers, but with more PTSD symptoms (β = 0.304, *p* < 0.05) and more depressive symptoms (β = 0.231, *p* < 0.05).

**Table 2:** Linear regression analyses predicting fitness, body composition, cardiovascular risk markers, and mental health outcomes with absolute grip strength scores, after controlling for sex, age and BMI.

	Sex	Age	BMI	Absolute GS	Total model
Cardiorespiratory fitness					
Estimated VO_2_max (in mL/kg/min)	− 0.340**	− 0.160*	− 0.297***	0.100	0.38***
Body composition					
Muscle mass (in %)	− 0.450***	0.024	− 0.577***	0.204***	0.89***
Fat mass (in %)	0.420***	− 0.012	0.612***	− 0.208***	0.89***
Cardiovascular risk markers					
Systolic blood pressure (in mm HG)	− 0.420***	0.134	0.240**	0.162	0.34***
Diastolic blood pressure (in mm HG)	− 0.310***	0.119	0.403***	0.033	0.26***
Total cholesterol (in mmol/l)	0.141	− 0.048	0.333**	0.056	0.13**
LDL cholesterol (in mmol/l)	0.111	− 0.078	0.248*	0.072	0.06
HDL cholesterol (in mmol/l)	0.139	− 0.103	− 0.038	− 0.241	0.14**
Triglycerides (in mmol/l)	− 0.130	0.255**	0.261**	0.081	0.20***
HbA1c (in %)	− 0.046	0.203*	0.352***	0.083	0.22***
Mental health outcomes					
PTSD symptoms (IES-R)	0.488***	0.304***	− 0.247**	0.304*	0.14***
Depressive symptoms (PHQ-9)	0.390**	0.286**	− 0.249*	0.231*	0.11**
Anxiety symptoms (GAD-7)	0.359**	0.301**	− 0.105	0.066	0.17***
Pain (VAS)	0.430**	0.248**	0.066	0.205	0.19***
Quality of life (WHO-5)	− 0.231	− 0.150	0.163	− 0.133	0.04

GS: Grip strength. VO_2_max: Maximal oxygen uptake. MVPA: Moderate-to-vigorous physical activity. LDL: Low density lipoprotein. HDL: High density lipoprotein. HbA1c: Glycated hemoglobin. IES-R: 22-item Index of Event Scale—revised. PHQ-9: 9-item depression scale of the Patient Health Questionnaire. GAD-7: 7-item General Anxiety Disorder Index. 7-item ISI: Insomnia Severity Index. VAS: 5-item Visual Analogue Scale. WHO-5: 5-item Quality of Life Index of the World Health Organization. *p < 0.05. **p < 0.01. ***p < 0.001.

Table 2 further shows that women had significantly lower VO_2_max, lower muscle mass, higher percentage body fat, and lower blood pressure. Moreover, women reported higher symptoms of PTSD, depression, anxiety, and pain, whereas they had lower ratings on quality of life. Age was negatively associated with VO_2_max, whereas a positive association existed with nearly all cardiovascular risk markers (with the exception of HDL cholesterol). Older participants also reported higher symptoms of PTSD, depression, anxiety and pain. Finally, BMI was negatively associated with VO_2_max and muscle mass, whereas positive associations existed with percentage body fat and nearly all cardiovascular risk markers (except HDL cholesterol). In turn, participants with higher BMI reported lower symptoms of PTSD and depression.

### Associations between normalized grip strength and health outcomes

**Figure 2:**
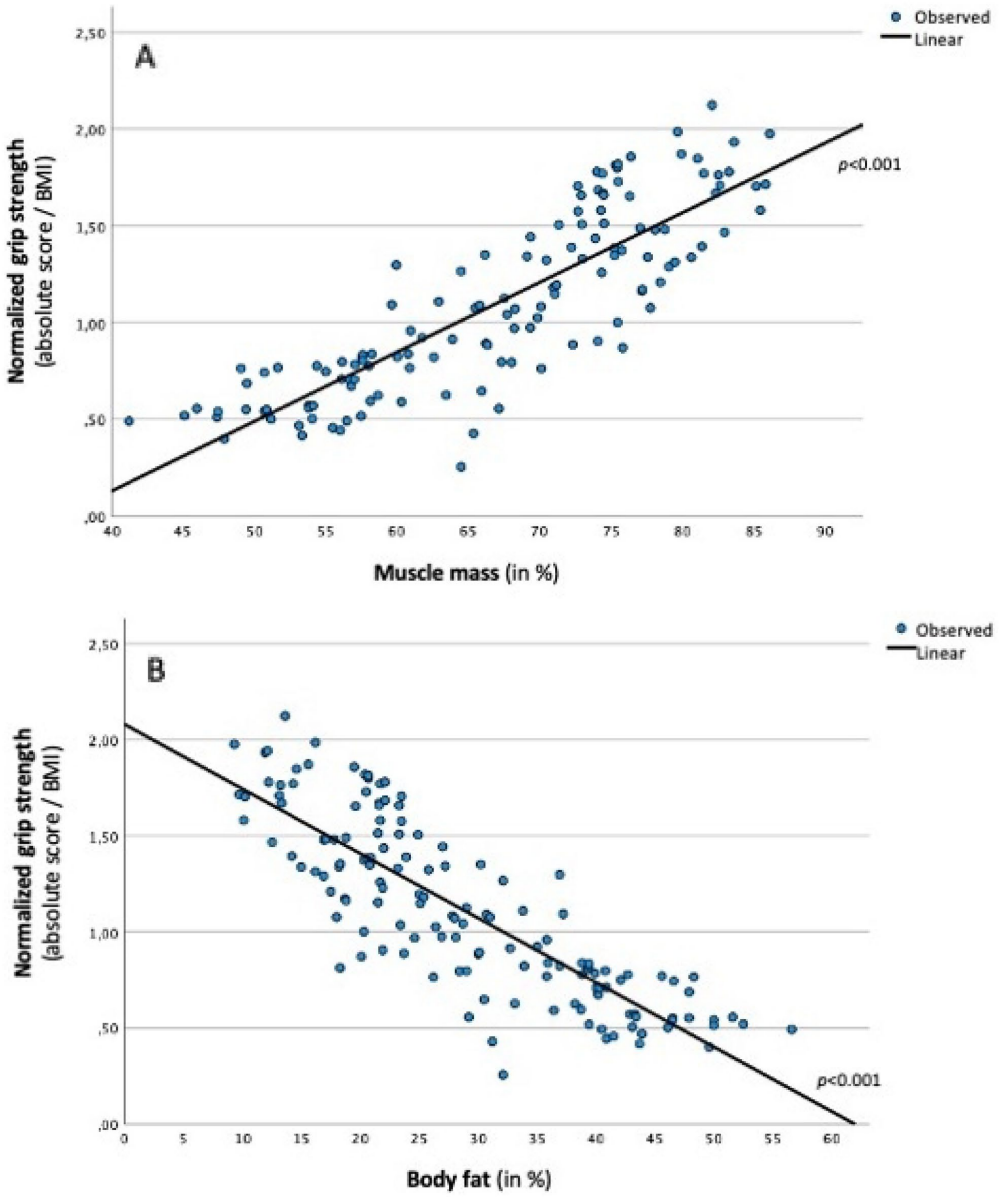
Association between normalized grip strength and percentage muscle mass (**A**) and body fat (**B**).

The pattern for normalized grip strength was similar to that described previously for the absolute values (Table 3), but some of the associations were stronger. Relatively strong associations were observed for both percentage muscle mass (β = 0.575, *p* < 0.001, Fig. 2A) and body fat (β = − 0.582, *p* < 0.001; Fig. 2B). Normalized grip strength was also positively associated with VO_2_max (β = 0.286, *p* < 0.05). Finally, higher normalized grip strength was associated with more symptoms of PTSD (β = 0.363, *p* < 0.05) and depression (β = 0.292, *p* < 0.05). No statistically significant associations were found for the remaining health outcomes.

**Table 3:** Linear regression analyses predicting fitness, body composition, cardiovascular risk markers, and mental health outcomes with normalized grip strength scores, after controlling for sex and age.

	Sex	Age	Normalized GS	Total model
Cardiorespiratory fitness				
Estimated VO_2_max (in mL/kg/min)	− 0.277*	− 0.220**	0.286*	0.34***
Body composition				
Muscle mass (in %)	− 0.307***	− 0.096*	0.575***	0.75***
Fat mass (in %)	0.273***	0.143**	− 0.582***	0.74***
Cardiovascular risk markers				
Systolic blood pressure (in mm HG)	− 0.495***	0.232**	− 0.005	0.28***
Diastolic blood pressure (in mm HG)	− 0.391**	0.250**	− 0.191	0.15***
Total cholesterol (in mmol/l)	− 0.013	0.045	− 0.241	0.06
LDL cholesterol (in mmol/l)	− 0.037	− 0.013	− 0.189	0.03
HDL cholesterol (in mmol/l)	0.170	− 0.153	− 0.198	0.13**
Triglycerides (in mmol/l)	− 0.193	0.349***	− 0.080	0.15***
HbA1c (in %)	− 0.088	0.321***	− 0.092	0.12**
Mental health outcomes				
PTSD symptoms (IES-R)	0.265**	0.469***	0.363*	0.12***
Depressive symptoms (PHQ-9)	0.373**	0.233**	0.292*	0.08**
Anxiety symptoms (GAD-7)	0.373**	0.281***	0.116	0.17***
Pain (VAS)	0.432**	0.310***	0.195	0.18***
Quality of life (WHO-5)	− 0.275	− 0.125	− 0.243	0.03

GS: Grip strength. VO_2_max: Maximal oxygen uptake. MVPA: Moderate-to-vigorous physical activity. LDL: Low density lipoprotein. HDL: High density lipoprotein. HbA1c: Glycated hemoglobin. IES-R: 22-item Index of Event Scale—revised. PHQ-9: 9-item depression scale of the Patient Health Questionnaire. GAD-7: 7-item General Anxiety Disorder Index. 7-item ISI: Insomnia Severity Index. VAS: 5-item Visual Analogue Scale. WHO-5: 5-item Quality of Life Index of the World Health Organization. *p < 0.05. **p < 0.01. ***p < 0.001.

### Differences in health outcomes between participants with low vs. higher grip strength

Table 4 confirms differences in body composition between participants with weak grip strength and their stronger counterparts, with the former having lower muscle mass and higher fat mass. Participants with weak grip strength also report lower scores for PTSD, depression and pain. No statistically significant differences were found for VO_2_max and the cardiovascular risk markers. Whereas the previously described sex differences were corroborated in the ANCOVAs, no statistically significant interactions were observed between grip strength and sex for any of the outcomes.

**Table 4:** Two-way analyses of covariance (controlled for age and BMI) with grip strength and sex as fixed factors, and fitness, body composition, cardiovascular risk markers, and mental health as outcome variables.

	Low GS				High GS									
	Men		Women		Men		Women		GS		Sex		GS x Sex	
	M	*SD*	*M*	*SD*	*M*	*SD*	*M*	*SD*	*F*	η^2^	*F*	η^2^	*F*	η^2^
Cardiorespiratory fitness														
Estimated VO_2_max (in mL/kg/min)	41.93	23.64	25.06	9.51	37.18	11.08	27.07	10.51	0.01	0.000	29.69***	0.205	1.44	0.012
Body composition														
Muscle mass (in %)	74.71	4.05	58.38	6.47	75.76	6.03	59.13	9.00	19.94***	0.135	298.38***	0.700	0.01	0.000
Fat mass (in %)	21.06	4.25	37.78	7.65	20.08	6.40	36.61	9.96	24.66***	0.153	306.848***	0.693	0.04	0.000
Cardiovascular risk markers														
Systolic blood pressure (in mm HG)	125.69	14.95	110.37	11.57	126.21	8.84	115.86	13.02	0.67	0.005	54.39***	0.286	2.30	0.017
Diastolic blood pressure (in mm HG)	81.41	9.27	78.97	7.13	83.80	6.65	79.69	10.30	0.05	0.000	15.37***	0.102	0.10	0.001
Total cholesterol (in mmol/l)	3.86	1.03	4.30	0.81	3.91	0.94	4.15	0.85	0.56	0.005	0.94	0.008	0.49	0.004
LDL cholesterol (in mmol/l)	2.01	0.71	2.31	0.75	2.14	0.65	2.22	0.72	0.03	0.000	1.88	0.015	0.59	0.005
HDL cholesterol (in mmol/l)	1.13	0.30	1.38	0.30	1.07	0.31	1.20	0.26	3.44	0.006	11.18***	0.085	1.12	0.009
Triglycerides (in mmol/l)	1.78	0.93	1.47	0.65	1.68	0.83	1.60	0.83	0.24	0.002	5.78*	0.046	0.49	0.004
HbA1c (in %)	5.31	0.17	5.37	0.38	5.37	0.24	5.36	0.34	0.19	0.001	1.53	0.012	0.17	0.001
Mental health outcomes														
PTSD symptoms (IES-R)	21.10	21.86	34.61	19.37	33.89	20.79	43.44	23.69	8.67**	0.060	11.87***	0.081	0.20	0.001
Depressive symptoms (PHQ-9)	6.92	4.81	10.61§	8.91	10.71	6.94	13.23	7.00	6.87**	0.048	8.33**	0.058	0.13	0.001
Anxiety symptoms (GAD-7)	5.95	4.71	10.76	6.46	8.18	6.58	12.27	6.34	2.51	0.012	13.51***	0.096	0.00	0.000
Pain (VAS)	10.88	10.62	30.64	23.49	24.06	21.36	36.49	20.01	5.49*	0.042	15.09***	0.107	0.48	0.004
Quality of life (WHO-5)	16.00	7.68	13.87	6.88	13.46	6.72	11.94	7.64	3.40	0.025	3.07	0.022	0.04	0.000

GS: Grip strength. VO_2_max: Maximal oxygen uptake. LDL: Low density lipoprotein. HDL: High density lipoprotein. HbA1c: Glycated hemoglobin. IES-R: 22-item Index of Event Scale—revised. PHQ-9: 9-item depression scale of the Patient Health Questionnaire. GAD-7: 7-item General Anxiety Disorder Index. 7-item ISI: Insomnia Severity Index. VAS: 5-item Visual Analogue Scale. WHO-5: 5-item Quality of Life Index of the World Health Organization. *p < 0.05. **p < 0.01. ***p < 0.001.

## Discussion

The key findings of the present study are that in a diverse sample of forcibly displaced people living in a Greek refugee camp, grip strength was positively associated with percentage muscle mass, whereas a negative association existed for percentage body fat. No statistically significant associations occurred between grip strength and cardiovascular risk markers. In contrast, we found that participants with higher normalized grip strength reported higher levels of PTSD and depressive symptoms.

Five hypotheses were formulated, and each of these will now be discussed separately. Our first hypothesis claimed that men would report higher grip strength than women. In line with previous research^24,25^, this hypothesis was supported and similarly strong effects were found for absolute and normalized grip strength scores (approximately 64% of explained variance).

In contrast, our second hypothesis, which suggested a curvilinear relationship between grip strength and age with a peak around the age of 40 years, was not supported by our empirical data. This contrasts with previous research in younger populations showing that muscle strength increases from childhood to younger adulthood due to changes in muscle mass and muscle fiber size^3^. Whereas we found an increase in grip strength until the age of 30 years, the quadratic regression model did not reach statistical significance. One reason for the absence of a statistically significant relationship might be that due to our inclusion criteria, all participants were between 16 and 59 years of age, whereas the age-related increase in grip strength is strongest during childhood and early adolescence^2^, and the strongest decline occurs after the age of 60 years^60,61^. Thus, excluding younger and older participants may have resulted in a relatively flat curve.

Our third hypothesis assumed a weak positive association between grip strength and cardiorespiratory fitness. This hypothesis was supported in the sense that higher VO_2_max was associated with higher normalized grip strength. However, no statistically significant relation was found for absolute grip strength, or when participants with weak grip strength were compared to their stronger counterparts. This is in line with the conceptual idea that muscle strength and cardiorespiratory fitness represent clearly distinguishable components of physical fitness^32^, and that muscular strength and cardiorespiratory fitness are independent predictors of health-related quality of life^62^ and mortality^63^. Our data also support previous research showing that after controlling for relevant confounders, only weak associations existed between grip strength and VO_2_max^33,61^.

Our fourth hypothesis focused on the association between grip strength and the various health outcomes. Our findings were mixed and did not fully support a general relation between these variables. Expectedly, higher grip strength was associated with higher relative muscle mass. This is in line with previous investigations showing that grip strength is a good indicator of both whole- and upper-body muscular strength^3,64^. In contrast, a negative association was observed between grip strength and percentage body fat, which corroborates previous research showing that overweight and obese people often achieve lower grip strength values^65^. This finding might seem paradoxical as overweight and obese people have greater (absolute) muscle mass, which in turn is associated with muscle strength^66,67^. Furthermore, overweight and obesity might induce a certain training effect via weight bearing, which can strengthen the muscles. However, this effect might be more obvious for the lower extremities^68^. On the flipside, overweight and obese people tend to engage in more sedentary activities and less physical activity^69,70^. Overweight and obesity may also induce pain, which can have a negative impact on the performance in muscle strength tests^71^. Studies also showed that there are differences in muscles between overweight/obese and normal weight people. Thus, while the former have fewer type I muscles, they seem to possess more type IIb muscle fibres^72^.

The fact that we did not observe any statistically significant associations between grip strength and the cardiovascular risk markers was unexpected and contrasts with a relatively large body of previous studies, in which higher grip strength scores were associated with more favourable cardiovascular risk profiles, fewer cardiovascular diseases and lower risk of cardiovascular mortality^37,38,73,74^. Accordingly, previous studies showed that lower grip strength is associated with higher risk of hypertension^75^, metabolic syndrome^30,38^, and diabetes^75,76^. A recent meta-analysis further showed that accounting for cardiovascular risk factors attenuated the relationship between grip strength and mortality^11^. The reason why this association was not supported in our sample is not clear. Our assumption is that other factors that were not systematically assessed in the present study (e.g. dietary behaviour) may have had a stronger influence on cardiovascular risk markers, and thus have overlaid the relationships observed in other populations.

Finally, prior investigations have shown close links between grip strength and mental health outcomes, indicating that people with higher grip strength report better general health perceptions^77^, better health-related quality of life^8,39^, and less depressive symptoms^7,78^. The associations with the above mental health outcomes have been attributed to stronger declines in physical functioning among people with lower grip strength through an association with adverse health behaviours (e.g., dietary behavior, physical activity), increased frailty, limitations in activities of daily living, and or social isolation^79^. It is also believed that muscle contraction increases the release of cytokines and myokines into the circulation, which in turn can decrease the risk of mental health issues^80^. Moreover, it has been argued that sarcopenia might be related with inflammatory profiles that are associated with an increased risk of mental health problems^80^. In marked contrast to these findings, our results showed that participants with higher grip strength reported more symptoms of PTSD and depression, whereas no statistically significant associations were found with anxiety and quality of life. Again, these relationships were unexpected, and we can only speculate about the underlying reasons. One possible explanation is that grip strength is associated with higher levels of sensation seeking^81^, which can lead to participants being more willing to take risks before and during the flight or while living in the refugee camp; this in turn, can increase their individual risk to be exposed to traumatic experiences. Alternatively, it could also be that people with higher grip strength have more confidence in their physical ability. Such people may feel capable of a more difficult flight, which in turn increases the risk of traumatic experiences. Accordingly, the underlying mechanism would not be an increased willingness to take risks, but rather a higher level of confidence in one's own (physical) abilities. A further alternative explanation could be that being stuck in a refugee camp is a frustrating (and sometimes traumatising) experience^82,83^. Since in Greece camp residents are not given the opportunity to pursue a gainful employment, the level of frustration might be particularly pronounced among residents who feel strong and fit.

Our fifth hypothesis was generally supported. Thus, the relationships between grip strength and the various health outcomes pointed in the same direction independent on whether absolute or normalized grip strength were used, or whether comparisons were made between participants with weak versus higher grip strength. In line with previous research^30^, however, our data showed that some associations were stronger for normalized scores. Currently, researchers still do not agree on whether grip strength should be adjusted for body mass. While some experts recommend this procedure^28,29^, others criticize that normalization might lead to overscaling and simply reverse what was a positive correlation between body mass and the scaled variable into a negative relationship^29^. However, such an inversion was not observed in the present sample, which corresponds well with UK Biobank data (based on more than 350′000 participants), where grip strength was associated with health outcomes, independent of whether grip strength was expressed in absolute (kg) or in relative terms (with reference to height/ weight, fat-free mass, BMI, Fat-free mass index, or as z-scores^40^.

The strengths of our paper are that we focused on a so-far under-researched vulnerable population, that both physical and mental health outcomes were addressed, and that grip strength was assessed repeatedly with both the dominant and non-dominant hand. Potential limitations are that some possible influences were not assessed, which were associated with grip strength in prior studies. These factors include socioeconomic status^84^, smoking^85^, dietary behaviour^86,87^, and time of the day^88^. Because our data are cross-sectional, we also acknowledge that no causal conclusions are possible^11^. While we applied grip strength cut-offs that were previously used with participants from LMICs, it is difficult to generalize grip strength norms across populations^2,89^. For instance, as shown by Dodds et al.^31^, grip strength scores are significantly higher in participants from industrialized to less industrialized countries. While we were able to control motivation during the assessment of cardiorespiratory fitness by using a sub-maximal fitness test, this was not possible for the grip strength test. Thus, although support for the internal consistency of the grip strength assessments was found, performance motivation might have influenced the results. Finally, we caution against a too broad generalization of the findings beyond our sample because forcibly displaced individuals constitute a diverse population, and because health risks among forcibly displaced people can vary based on numerous sociodemographic and structural determinants, including behavioral factors, origin, migration status, duration of residence, living conditions, migration policies, and access to health services^90,91^.

From a practical point of view, measuring muscle strength in large populations can be complex and costly^77^, whereas the application of the grip strength test is non-invasive, simple to implement, inexpensive and reliable^92,93^. Accordingly, assessments are quickly obtainable by a range of different health professionals^94^, and hand grip strength is often used in diagnostic algorithms for sarcopenia^77^. Additionally, the grip strength test can not only be used in adults, but also proved to be a suitable indicator for musculoskeletal fitness in children^95,96^. Accordingly, the assessment of handgrip strength is recommended by the American Academy of Medicine^97^ as part of school-based fitness testing and as a screening tool for adequate levels of muscle strength for bone health. Moreover, public health recommendations suggest that forcibly displaced people should engage in muscle-strengthening activities at least two hours per week. Accordingly, intervention policies should be formulated in a way so that they encourage weight training in both men and women^74^. Given this background, grip strength has the potential to be used as an easy-to-implement measure to assess changes in upper-body muscle strength in resource-limited settings.

## Conclusions

Measuring grip strength in forcibly displaced people can be a useful way to assess their overall muscle strength and body composition. Grip strength tests are easy to implement, and results can be used to assess the effects of specific intervention measures. Nevertheless, our results question the usefulness of grip strength as a marker of cardiovascular health and mental wellbeing in a refugee camp setting.

### Supplementary Information

Supplementary Information 1.

Supplementary Information 2.

## Data Availability

The datasets used and analyzed during the current study are available as supplementary online material (SPSS file and Syntax).

## Abbreviations

ANCOVAs: Analyses of covariance
BMI: Body mass index
DSM-5: Diagnostic and statistical manual of mental disorders 5
GAD-7: General Anxiety Disorder scale
HbA1c: Glygated hemoglobin
HDL: High-density lipoprotein
ICD-10: International classification of diseases, 10th revision, of the World Health Organization
IES-R: Impact of Event Scale-Revised
Kurt: Kurtosis
LDL: Low-density lipoprotein
LMICs: Low-to-middle-income countries
PAC: Point-of-care
PHQ-9: Patient Health Questionnaire
PTSD: Post-traumatic stress disorder
RCT: Randomized controlled trial
Skew: Skewness
SPSS: Statistical Package for the Social Sciences
VAS: Visual Analogue Scale
VIF: Variance inflation factor
VO_2_max: Maximum oxygen uptake
WHO: World Health Organization
