# Supplementary material for: Associations between grip strength, cardiorespiratory fitness, cardiovascular risk and mental health in forcibly displaced people from a Greek refugee camp
Source: Sci Rep. 2023 Nov 28;13:20970. doi: 10.1038/s41598-023-48032-5 (PMC10684690; doi:10.1038/s41598-023-48032-5)
Supplement: Supplementary file 2 — Supplementary Information 2. [file 41598_2023_48032_MOESM2_ESM.docx]

**SPSS Syntax used for calculations in the manuscript entitled "Associations between grip strength, cardiorespiratory fitness, cardiovascular risk and mental health in forcibly displaced people from a Greek refugee camp"**

USE ALL.

COMPUTE filter_$=(hand_tot_T1 > 0 & age >= 1).

VARIABLE LABELS filter_$ 'hand_tot_T1 > 0 & age >= 1 (FILTER)'.

VALUE LABELS filter_$ 0 'Not Selected' 1 'Selected'.

FORMATS filter_$ (f1.0).

FILTER BY filter_$.

EXECUTE.

Text: Sample characteristics

******************************************************************************************

FREQUENCIES VARIABLES=sex age lang country education

/ORDER=ANALYSIS.

DESCRIPTIVES VARIABLES=age Height_T1 Weight_T1 BMI_T1 months_away months_camp

/STATISTICS=MEAN STDDEV MIN MAX SKEWNESS KURTOSIS.

******************************************************************************************

Table 1: Descriptive statistics

******************************************************************************************

DESCRIPTIVES VARIABLES=hand_tot_T1 GS_BMI VO2max_T1 Muscle_percentage_T1 Fat_percentage_T1

BloodPressure_H_tot_T1 BloodPressure_L_tot_T1 Chol_T1 LDL_T1 HDL_T1 TG_T1 HbA1c_T1

IES_tot_T1 PHQ_tot_T1 GAD_tot_T1 VAS_tot_T1 WHO_tot_T1

/STATISTICS=MEAN STDDEV MIN MAX KURTOSIS SKEWNESS.

******************************************************************************************Associations with sex

******************************************************************************************

UNIANOVA hand_tot_T1 BY sex WITH age bmi_t1

/METHOD=SSTYPE(3)

/INTERCEPT=INCLUDE

/PRINT ETASQ DESCRIPTIVE

/CRITERIA=ALPHA(.05)

/DESIGN=age bmi_t1 sex.

UNIANOVA GS_BMI BY sex WITH age

/METHOD=SSTYPE(3)

/INTERCEPT=INCLUDE

/PRINT ETASQ DESCRIPTIVE

/CRITERIA=ALPHA(.05)

/DESIGN= age sex.

CROSSTABS

/TABLES=sex BY hand_tot_kat

/FORMAT=AVALUE TABLES

/STATISTICS=CHISQ CC

/CELLS=COUNT ROW COLUMN

/COUNT ROUND CELL.

******************************************************************************************

Figure 1: Associations with age

******************************************************************************************

DATASET ACTIVATE DataSet1.

REGRESSION

/MISSING LISTWISE

/STATISTICS COEFF OUTS CI(95) R ANOVA COLLIN TOL CHANGE

/CRITERIA=PIN(.05) POUT(.10)

/NOORIGIN

/DEPENDENT hand_tot_T1

/METHOD=ENTER sex bmi_t1 age

/METHOD=ENTER sex bmi_t1 age.

DATASET ACTIVATE DataSet1.

REGRESSION

/MISSING LISTWISE

/STATISTICS COEFF OUTS CI(95) R ANOVA COLLIN TOL CHANGE

/CRITERIA=PIN(.05) POUT(.10)

/NOORIGIN

/DEPENDENT GS_BMI

/METHOD=ENTER sex bmi_t1 age

/METHOD=ENTER sex bmi_t1 age.

* Kurvenanpassung.

TSET NEWVAR=NONE.

CURVEFIT

/VARIABLES=hand_tot_T1 WITH age

/CONSTANT

/MODEL=LINEAR QUADRATIC

/PLOT FIT.

* Kurvenanpassung.

TSET NEWVAR=NONE.

CURVEFIT

/VARIABLES=GS_BMI WITH age

/CONSTANT

/MODEL=LINEAR QUADRATIC

/PLOT FIT.

UNIANOVA age BY hand_tot_kat WITH sex bmi_t1

/METHOD=SSTYPE(3)

/INTERCEPT=INCLUDE

/PRINT ETASQ DESCRIPTIVE

/CRITERIA=ALPHA(.05)

/DESIGN=sex bmi_t1 hand_tot_kat.

******************************************************************************************Associations with BMI

******************************************************************************************

DATASET ACTIVATE DataSet1.

REGRESSION

/MISSING LISTWISE

/STATISTICS COEFF OUTS CI(95) R ANOVA COLLIN TOL CHANGE

/CRITERIA=PIN(.05) POUT(.10)

/NOORIGIN

/DEPENDENT hand_tot_T1

/METHOD=ENTER sex age bmi_t1

/METHOD=ENTER sex age bmi_t1.

DATASET ACTIVATE DataSet1.

REGRESSION

/MISSING LISTWISE

/STATISTICS COEFF OUTS CI(95) R ANOVA COLLIN TOL CHANGE

/CRITERIA=PIN(.05) POUT(.10)

/NOORIGIN

/DEPENDENT GS_BMI

/METHOD=ENTER sex age bmi_t1

/METHOD=ENTER sex age bmi_t1 .

UNIANOVA BMI_t1 BY hand_tot_kat WITH sex age

/METHOD=SSTYPE(3)

/INTERCEPT=INCLUDE

/PRINT ETASQ DESCRIPTIVE

/CRITERIA=ALPHA(.05)

/DESIGN=sex age hand_tot_kat.

******************************************************************************************

Table 2: Regressionsanalysen GS (absolute score), controlled for sex, age and BMI

******************************************************************************************

DATASET ACTIVATE DataSet1.

REGRESSION

/MISSING LISTWISE

/STATISTICS COEFF OUTS CI(95) R ANOVA COLLIN TOL CHANGE

/CRITERIA=PIN(.05) POUT(.10)

/NOORIGIN

/DEPENDENT VO2max_T1

/METHOD=ENTER sex age bmi_t1

/METHOD=ENTER sex age bmi_t1 hand_tot_T1.

DATASET ACTIVATE DataSet1.

REGRESSION

/MISSING LISTWISE

/STATISTICS COEFF OUTS CI(95) R ANOVA COLLIN TOL CHANGE

/CRITERIA=PIN(.05) POUT(.10)

/NOORIGIN

/DEPENDENT Muscle_percentage_t1

/METHOD=ENTER sex age bmi_t1

/METHOD=ENTER sex age bmi_t1 hand_tot_T1.

DATASET ACTIVATE DataSet1.

REGRESSION

/MISSING LISTWISE

/STATISTICS COEFF OUTS CI(95) R ANOVA COLLIN TOL CHANGE

/CRITERIA=PIN(.05) POUT(.10)

/NOORIGIN

/DEPENDENT Fat_percentage_T1

/METHOD=ENTER sex age bmi_t1

/METHOD=ENTER sex age bmi_t1 hand_tot_T1.

DATASET ACTIVATE DataSet1.

REGRESSION

/MISSING LISTWISE

/STATISTICS COEFF OUTS CI(95) R ANOVA COLLIN TOL CHANGE

/CRITERIA=PIN(.05) POUT(.10)

/NOORIGIN

/DEPENDENT BloodPressure_H_tot_T1

/METHOD=ENTER sex age bmi_t1

/METHOD=ENTER sex age bmi_t1 hand_tot_T1.

DATASET ACTIVATE DataSet1.

REGRESSION

/MISSING LISTWISE

/STATISTICS COEFF OUTS CI(95) R ANOVA COLLIN TOL CHANGE

/CRITERIA=PIN(.05) POUT(.10)

/NOORIGIN

/DEPENDENT BloodPressure_L_tot_T1

/METHOD=ENTER sex age bmi_t1

/METHOD=ENTER sex age bmi_t1 hand_tot_T1.

DATASET ACTIVATE DataSet1.

REGRESSION

/MISSING LISTWISE

/STATISTICS COEFF OUTS CI(95) R ANOVA COLLIN TOL CHANGE

/CRITERIA=PIN(.05) POUT(.10)

/NOORIGIN

/DEPENDENT Chol_T1

/METHOD=ENTER sex age bmi_t1

/METHOD=ENTER sex age bmi_t1 hand_tot_T1.

DATASET ACTIVATE DataSet1.

REGRESSION

/MISSING LISTWISE

/STATISTICS COEFF OUTS CI(95) R ANOVA COLLIN TOL CHANGE

/CRITERIA=PIN(.05) POUT(.10)

/NOORIGIN

/DEPENDENT LDL_T1

/METHOD=ENTER sex age bmi_t1

/METHOD=ENTER sex age bmi_t1 hand_tot_T1.

DATASET ACTIVATE DataSet1.

REGRESSION

/MISSING LISTWISE

/STATISTICS COEFF OUTS CI(95) R ANOVA COLLIN TOL CHANGE

/CRITERIA=PIN(.05) POUT(.10)

/NOORIGIN

/DEPENDENT HDL_T1

/METHOD=ENTER sex age bmi_t1

/METHOD=ENTER sex age bmi_t1 hand_tot_T1.

DATASET ACTIVATE DataSet1.

REGRESSION

/MISSING LISTWISE

/STATISTICS COEFF OUTS CI(95) R ANOVA COLLIN TOL CHANGE

/CRITERIA=PIN(.05) POUT(.10)

/NOORIGIN

/DEPENDENT TG_T1

/METHOD=ENTER sex age bmi_t1

/METHOD=ENTER sex age bmi_t1 hand_tot_T1.

DATASET ACTIVATE DataSet1.

REGRESSION

/MISSING LISTWISE

/STATISTICS COEFF OUTS CI(95) R ANOVA COLLIN TOL CHANGE

/CRITERIA=PIN(.05) POUT(.10)

/NOORIGIN

/DEPENDENT HbA1c_T1

/METHOD=ENTER sex age bmi_t1

/METHOD=ENTER sex age bmi_t1 hand_tot_T1.

DATASET ACTIVATE DataSet1.

REGRESSION

/MISSING LISTWISE

/STATISTICS COEFF OUTS CI(95) R ANOVA COLLIN TOL CHANGE

/CRITERIA=PIN(.05) POUT(.10)

/NOORIGIN

/DEPENDENT IES_tot_T1

/METHOD=ENTER sex age bmi_t1

/METHOD=ENTER sex age bmi_t1 hand_tot_T1.

DATASET ACTIVATE DataSet1.

REGRESSION

/MISSING LISTWISE

/STATISTICS COEFF OUTS CI(95) R ANOVA COLLIN TOL CHANGE

/CRITERIA=PIN(.05) POUT(.10)

/NOORIGIN

/DEPENDENT PHQ_tot_T1

/METHOD=ENTER sex age bmi_t1

/METHOD=ENTER sex age bmi_t1 hand_tot_T1.

DATASET ACTIVATE DataSet1.

REGRESSION

/MISSING LISTWISE

/STATISTICS COEFF OUTS CI(95) R ANOVA COLLIN TOL CHANGE

/CRITERIA=PIN(.05) POUT(.10)

/NOORIGIN

/DEPENDENT GAD_tot_T1

/METHOD=ENTER sex age bmi_t1

/METHOD=ENTER sex age bmi_t1 hand_tot_T1.

DATASET ACTIVATE DataSet1.

REGRESSION

/MISSING LISTWISE

/STATISTICS COEFF OUTS CI(95) R ANOVA COLLIN TOL CHANGE

/CRITERIA=PIN(.05) POUT(.10)

/NOORIGIN

/DEPENDENT VAS_tot_T1

/METHOD=ENTER sex age bmi_t1

/METHOD=ENTER sex age bmi_t1 hand_tot_T1.

DATASET ACTIVATE DataSet1.

REGRESSION

/MISSING LISTWISE

/STATISTICS COEFF OUTS CI(95) R ANOVA COLLIN TOL CHANGE

/CRITERIA=PIN(.05) POUT(.10)

/NOORIGIN

/DEPENDENT WHO_tot_T1

/METHOD=ENTER sex age bmi_t1

/METHOD=ENTER sex age bmi_t1 hand_tot_T1.

******************************************************************************************

Table 3: Regressionsanalysen Normalized GS, controlled for sex and age

******************************************************************************************

DATASET ACTIVATE DataSet1.

REGRESSION

/MISSING LISTWISE

/STATISTICS COEFF OUTS CI(95) R ANOVA COLLIN TOL CHANGE

/CRITERIA=PIN(.05) POUT(.10)

/NOORIGIN

/DEPENDENT VO2max_T1

/METHOD=ENTER sex age

/METHOD=ENTER sex age GS_BMI.

DATASET ACTIVATE DataSet1.

REGRESSION

/MISSING LISTWISE

/STATISTICS COEFF OUTS CI(95) R ANOVA COLLIN TOL CHANGE

/CRITERIA=PIN(.05) POUT(.10)

/NOORIGIN

/DEPENDENT Muscle_percentage_t1

/METHOD=ENTER sex age

/METHOD=ENTER sex age GS_BMI.

DATASET ACTIVATE DataSet1.

REGRESSION

/MISSING LISTWISE

/STATISTICS COEFF OUTS CI(95) R ANOVA COLLIN TOL CHANGE

/CRITERIA=PIN(.05) POUT(.10)

/NOORIGIN

/DEPENDENT Fat_percentage_T1

/METHOD=ENTER sex age

/METHOD=ENTER sex age GS_bmi.

DATASET ACTIVATE DataSet1.

REGRESSION

/MISSING LISTWISE

/STATISTICS COEFF OUTS CI(95) R ANOVA COLLIN TOL CHANGE

/CRITERIA=PIN(.05) POUT(.10)

/NOORIGIN

/DEPENDENT BloodPressure_H_tot_T1

/METHOD=ENTER sex age

/METHOD=ENTER sex age GS_BMI.

DATASET ACTIVATE DataSet1.

REGRESSION

/MISSING LISTWISE

/STATISTICS COEFF OUTS CI(95) R ANOVA COLLIN TOL CHANGE

/CRITERIA=PIN(.05) POUT(.10)

/NOORIGIN

/DEPENDENT BloodPressure_L_tot_T1

/METHOD=ENTER sex age

/METHOD=ENTER sex age GS_BMI.

DATASET ACTIVATE DataSet1.

REGRESSION

/MISSING LISTWISE

/STATISTICS COEFF OUTS CI(95) R ANOVA COLLIN TOL CHANGE

/CRITERIA=PIN(.05) POUT(.10)

/NOORIGIN

/DEPENDENT Chol_T1

/METHOD=ENTER sex age

/METHOD=ENTER sex age GS_BMI.

DATASET ACTIVATE DataSet1.

REGRESSION

/MISSING LISTWISE

/STATISTICS COEFF OUTS CI(95) R ANOVA COLLIN TOL CHANGE

/CRITERIA=PIN(.05) POUT(.10)

/NOORIGIN

/DEPENDENT LDL_T1

/METHOD=ENTER sex age

/METHOD=ENTER sex age GS_BMI.

DATASET ACTIVATE DataSet1.

REGRESSION

/MISSING LISTWISE

/STATISTICS COEFF OUTS CI(95) R ANOVA COLLIN TOL CHANGE

/CRITERIA=PIN(.05) POUT(.10)

/NOORIGIN

/DEPENDENT HDL_T1

/METHOD=ENTER sex age

/METHOD=ENTER sex age GS_BMI.

DATASET ACTIVATE DataSet1.

REGRESSION

/MISSING LISTWISE

/STATISTICS COEFF OUTS CI(95) R ANOVA COLLIN TOL CHANGE

/CRITERIA=PIN(.05) POUT(.10)

/NOORIGIN

/DEPENDENT TG_T1

/METHOD=ENTER sex age

/METHOD=ENTER sex age GS_BMI.

DATASET ACTIVATE DataSet1.

REGRESSION

/MISSING LISTWISE

/STATISTICS COEFF OUTS CI(95) R ANOVA COLLIN TOL CHANGE

/CRITERIA=PIN(.05) POUT(.10)

/NOORIGIN

/DEPENDENT HbA1c_T1

/METHOD=ENTER sex age

/METHOD=ENTER sex age GS_BMI.

DATASET ACTIVATE DataSet1.

REGRESSION

/MISSING LISTWISE

/STATISTICS COEFF OUTS CI(95) R ANOVA COLLIN TOL CHANGE

/CRITERIA=PIN(.05) POUT(.10)

/NOORIGIN

/DEPENDENT IES_tot_T1

/METHOD=ENTER age sex

/METHOD=ENTER age sex GS_BMI.

DATASET ACTIVATE DataSet1.

REGRESSION

/MISSING LISTWISE

/STATISTICS COEFF OUTS CI(95) R ANOVA COLLIN TOL CHANGE

/CRITERIA=PIN(.05) POUT(.10)

/NOORIGIN

/DEPENDENT PHQ_tot_T1

/METHOD=ENTER sex age

/METHOD=ENTER sex age GS_BMI.

DATASET ACTIVATE DataSet1.

REGRESSION

/MISSING LISTWISE

/STATISTICS COEFF OUTS CI(95) R ANOVA COLLIN TOL CHANGE

/CRITERIA=PIN(.05) POUT(.10)

/NOORIGIN

/DEPENDENT GAD_tot_T1

/METHOD=ENTER sex age

/METHOD=ENTER sex age GS_BMI.

DATASET ACTIVATE DataSet1.

REGRESSION

/MISSING LISTWISE

/STATISTICS COEFF OUTS CI(95) R ANOVA COLLIN TOL CHANGE

/CRITERIA=PIN(.05) POUT(.10)

/NOORIGIN

/DEPENDENT VAS_tot_T1

/METHOD=ENTER sex age

/METHOD=ENTER sex age GS_BMI.

DATASET ACTIVATE DataSet1.

REGRESSION

/MISSING LISTWISE

/STATISTICS COEFF OUTS CI(95) R ANOVA COLLIN TOL CHANGE

/CRITERIA=PIN(.05) POUT(.10)

/NOORIGIN

/DEPENDENT WHO_tot_T1

/METHOD=ENTER sex age

/METHOD=ENTER sex age GS_BMI.

******************************************************************************************

Table 4: Differences between participants with low vs. high grip strength, after controlling for sex

******************************************************************************************

UNIANOVA VO2max_T1 BY hand_tot_kat sex WITH age bmi_t1

/METHOD=SSTYPE(3)

/INTERCEPT=INCLUDE

/PRINT ETASQ DESCRIPTIVE

/CRITERIA=ALPHA(.05)

/DESIGN=age bmi_t1 hand_tot_kat sex hand_tot_kat*sex.

UNIANOVA Muscle_percentage_T1 BY hand_tot_kat sex WITH age bmi_t1

/METHOD=SSTYPE(3)

/INTERCEPT=INCLUDE

/PRINT ETASQ DESCRIPTIVE

/CRITERIA=ALPHA(.05)

/DESIGN=age bmi_t1 hand_tot_kat sex hand_tot_kat*sex.

UNIANOVA Fat_percentage_T1 BY hand_tot_kat sex WITH age bmi_t1

/METHOD=SSTYPE(3)

/INTERCEPT=INCLUDE

/PRINT ETASQ DESCRIPTIVE

/CRITERIA=ALPHA(.05)

/DESIGN=age bmi_t1 hand_tot_kat sex hand_tot_kat*sex.

UNIANOVA BloodPressure_H_tot_T1 BY hand_tot_kat sex WITH age bmi_t1

/METHOD=SSTYPE(3)

/INTERCEPT=INCLUDE

/PRINT ETASQ DESCRIPTIVE

/CRITERIA=ALPHA(.05)

/DESIGN=age bmi_t1 hand_tot_kat sex hand_tot_kat*sex.

UNIANOVA BloodPressure_L_tot_T1 BY hand_tot_kat sex WITH age bmi_t1

/METHOD=SSTYPE(3)

/INTERCEPT=INCLUDE

/PRINT ETASQ DESCRIPTIVE

/CRITERIA=ALPHA(.05)

/DESIGN=age bmi_t1 hand_tot_kat sex hand_tot_kat*sex.

UNIANOVA Chol_T1 BY hand_tot_kat sex WITH age bmi_t1

/METHOD=SSTYPE(3)

/INTERCEPT=INCLUDE

/PRINT ETASQ DESCRIPTIVE

/CRITERIA=ALPHA(.05)

/DESIGN=age bmi_t1 hand_tot_kat sex hand_tot_kat*sex.

UNIANOVA LDL_T1 BY hand_tot_kat sex WITH age

/METHOD=SSTYPE(3)

/INTERCEPT=INCLUDE

/PRINT ETASQ DESCRIPTIVE

/CRITERIA=ALPHA(.05)

/DESIGN=age hand_tot_kat sex hand_tot_kat*sex.

UNIANOVA HDL_T1 BY hand_tot_kat sex With age bmi_t1

/METHOD=SSTYPE(3)

/INTERCEPT=INCLUDE

/PRINT ETASQ DESCRIPTIVE

/CRITERIA=ALPHA(.05)

/DESIGN= age bmi_t1 hand_tot_kat sex hand_tot_kat*sex.

UNIANOVA TG_T1 BY hand_tot_kat sex WITH age bmi_t1

/METHOD=SSTYPE(3)

/INTERCEPT=INCLUDE

/PRINT ETASQ DESCRIPTIVE

/CRITERIA=ALPHA(.05)

/DESIGN=age bmi_t1 hand_tot_kat sex hand_tot_kat*sex.

UNIANOVA HbA1c_T1 BY hand_tot_kat sex WITH age bmi_t1

/METHOD=SSTYPE(3)

/INTERCEPT=INCLUDE

/PRINT ETASQ DESCRIPTIVE

/CRITERIA=ALPHA(.05)

/DESIGN=age bmi_t1 hand_tot_kat sex hand_tot_kat*sex.

UNIANOVA IES_tot_T1 BY hand_tot_kat sex WITH age bmi_t1

/METHOD=SSTYPE(3)

/INTERCEPT=INCLUDE

/PRINT ETASQ DESCRIPTIVE

/CRITERIA=ALPHA(.05)

/DESIGN=age bmi_t1 hand_tot_kat sex hand_tot_kat*sex.

UNIANOVA PHQ_tot_T1 BY hand_tot_kat sex WITH age bmi_t1

/METHOD=SSTYPE(3)

/INTERCEPT=INCLUDE

/PRINT ETASQ DESCRIPTIVE

/CRITERIA=ALPHA(.05)

/DESIGN=age bmi_t1 hand_tot_kat sex hand_tot_kat*sex.

UNIANOVA GAD_tot_T1 BY hand_tot_kat sex WITH age bmi_t1

/METHOD=SSTYPE(3)

/INTERCEPT=INCLUDE

/PRINT ETASQ DESCRIPTIVE

/CRITERIA=ALPHA(.05)

/DESIGN=age bmi_t1 hand_tot_kat sex hand_tot_kat*sex.

UNIANOVA VAS_tot_T1 BY hand_tot_kat sex WITH age bmi_t1

/METHOD=SSTYPE(3)

/INTERCEPT=INCLUDE

/PRINT ETASQ DESCRIPTIVE

/CRITERIA=ALPHA(.05)

/DESIGN=age hand_tot_kat sex hand_tot_kat*sex.

UNIANOVA WHO_tot_T1 BY hand_tot_kat sex WITH age bmi_t1

/METHOD=SSTYPE(3)

/INTERCEPT=INCLUDE

/PRINT ETASQ DESCRIPTIVE

/CRITERIA=ALPHA(.05)

/DESIGN=age bmi_t1 hand_tot_kat sex hand_tot_kat*sex.
